# Prophylaxis of the Baby Welfare Station

**Published:** 1915-10

**Authors:** Frank E. Brundage

**Affiliations:** Buffalo


### BUFFALO MEDICAL JOURNAL

Yearly Volume 71 OCTOBER, 1915 No. 3

### ORIGINAL ARTICLES

The right is reserved to decline papers not dealing with practical medical and surgical subjects, and such as might offend or fail to interest readers. Contributors are solely responsible for opinions, methods of expression and revision of proof.

### Prophylaxis of the Baby Welfare Station.

FRANK E. BRUNDAGE, A. B., M. D., Buffalo.

Aside from the general care of the baby and utensils as taught by the nurse in the home, there are points of emphasis which essentially belong to the physician in charge of a milk station. Our main object is to keep the baby well and to do this we must not dismiss the mother by relying on her judgment concerning her babe's health. By strict questioning something is found to be wrong. False impressions must be corrected and advice for the future given in order to prevent unnecessary sickness. If we do not follow this procedure the place is merely a milk dispensary and not a prophylactic station as is intended.

For success in our work it is essential that we have a mother who is receptive and wants to learn. Those who follow directions usually get the best results and become a credit to herself and the station as well as benefiting the life of the babe. There are many however who after receiving the advice are not enough concerned about the health of the babe to carry out the instructions and it is only by the persistent effort on the part of the nurse who makes regular visits at the home that anything is accomplished. Besides having a receptive mother, regular attendance at the station is very necessary, at least every two weeks. In this way close attention may be given to the food and habits of the babe.

To be more definite, a discussion of the more important topics are in order. A breast fed baby being our ideal, every effort is made to maintain the breast supply. If the milk begins to diminish the symptoms of hunger and stationary weight are present. The mother is advised to drink a liberal supply of nourishing gruels. Her diet and exercise is regulated according to her particular need. If constipation is present, every means is taken to remedy the condition. A strict diet of fruit and vegetables with a small amount of meat, no white bread or potatoes will often accomplish much and seldom cause any trouble with the infant. Other details in regard to the mother's health which might affect the baby arises from time to time and are treated accordingly.

Hours of feeding is constantly abused. The greatest difficulty is met in convincing the mother that to establish regular hours of feeding is not only conducive to the best health of the babe but will materially lessen her trouble and work. I still believe in the majority of cases that the two hour feeding in bottle fed babies is proper. The second month a two and one half hour feeding and after the third month a three hour feeding. A normal breast fed baby may be started on a three hour feeding with usually good results. Some breast fed babies, not all, will do well, if not better, on longer intervals but to make it a general rule is asking too much of the mother for the actual benefit to be derived. When there are symptoms of over feeding or intestinal trouble lengthening of the intervals is always indicated, no matter what the age. These hours can readily be changed if there is regular attendance at the station.

Strength of food is the great problem. The formulas should be increased so gradually that no intestinal disturbance takes place. If a child is allowed to go for many weeks on one formula and is then suddenly changed to a much stronger mixture, trouble is encountered at once. A slight advance both as regards quality and quantity should be made every second to fourth week, depending upon the condition of the child.

Constipation in a babe is not in my estimation a normal condition. When not relieved, rectal fissure, piles and other local trouble is the result as well as having an irritable, bad sleeping baby. At the first suggestion of this trouble, the food is made as laxative as possible. Orange juice is given as early as three months if tolerated. Oatmeal gruel may be given either with the milk or separately. Milk sugar is always the sugar of choice in this condition. Milk of Magnesia or cascara is given as a last resort as the bowel becomes easily accustomed to the added stimulus. Many infants who are especially obstinate get good results when a little solid food is added to the diet. The condition of every babe's bowels should be ascertained at every visit to the station.

Nipple abuse is wide spread. Mothers give them for the most part to keep the baby quiet. It is fortunate many times that the baby has more sense than the mother and refuses to have it in their mouth. When a baby enters the station with this habit or contracts it afterward the mother is told the reason she should not continue the habit. The production of sore mouths, distorted features and tendency to adenoids is impressed on the mother as end results of this practice and it is usually sufficient to convince her of the bad practice.

Skin hygiene needs a great deal of attention, eczema, urticaria, erythema, ferunculosis, ringworm, impetigo and scabies are the common conditions encountered. The majority of these are a result of poor care. Internal and external treatment is given according to indication. Eczema which stands first is often due to the abuse of soap and water on a sensitive skin, combined with neglect or ignorance in the use of food and clothing. In many normal breast and bottle fed babies this condition is apparently a skin reaction. These are very obstinate to treat and take many months to cure with the best hygiene. It is more prevalent in the fat babies and those undernourished. If the diet is at fault a correction will often alleviate or cure entirely. Bran baths are often efficacious in many cases but in some no water at all is indicated. The skin is cleaned by some kind of oil.

Mouth conditions confine themselves to two conditions, Soor and Apthous stomatitis. These we find are due to uncleanliness in the breast or bottle fed. Some become so severe as to prevent feeding except by medicine dropper for several days. If the mouth is kept clean with a boric acid solution and a daily application of a silver nitrate solution, the condition rapidly clears up.

Solid food given early is one of the greatest temptations of most every mother. She is told that when the time comes a suitable diet will be given, otherwise nothing but milk. Most babies suffer from overfeeding at all periods but more especially about the eighth month. At this time the baby craves other food besides the milk and actually needs it for growth. The trouble lies in the fact that the mothers start in with too strong food and too much of it.

Adenoids and enlarged tonsils develop before our very eyes Many times when a child is not doing well, takes its nourishment poorly, is restless and has a continual discharge from the nose, the fault lies not in the diet but the presence of these abnormal growths. We advise them removed at once no matter what time of year if they are interfering with the child's growth.

When a baby is ill, I am convinced that in the majority of cases mechanical means and general care are of more value than any medicine. Heavy cough syrups, soothing syrups, colic cures and worm powders help usually the mothers mind only and does the child no good. We condemn the most of them and lay stress on the general good resistance of the babe to overcome disease.

Finally then, the success of a prophylactic milk station or welfare station depends not so much on the numbers seen but upon the careful attention of details in each case, given in time and carried out with a reasonable amount of precision.

---

**Amniotic Membrane in the Prevention of Peritoneal Adhesions.** C. B. Lyman, Denver, Col. Med., Aug. Salt solution is regarded as too quickly absorbed to be of benefit and the author is convinced of the inefficiency of mineral oil from experience with a case in which adhesions twice reformed after operations. Four cases are mentioned as showing good results from the use of amniotic membrane. The membrane is washed free from blood and placed in 1-2 per cent. formaldehyde in 70 per cent. alcohol and kept in the ice chest. An hour before using, it is transferred to physiologic salt solution. Very fine silk is used in a cambric needle. Split silk, twisted before using has proved convenient.

---

**Cotton Seed Dermatitis.** J. A. Nixon, Bristol, Eng., Bristol Medico-Chi. Jour., June, states that this occurs in "dockers" a word which scarcely requires translation, as it is really much more sensible than the American term longshoremen—handling cargoes of cotton. Itching occurs for several hours before the appearance of a rash, similar to that caused by mosquito bites. Some of the spots develop blebs. The rash disappears within a week unless there is further exposure or reinfection from scratching. Nixon has found a parasite which he believes to be identical with or very similar to pediculoides ventricosus, the cause of barley itch.

---

**Cheap Fire Grenades.** La Nature gives the following formulae: Calcium chlorid 157, Magnesium chlorid 56, Water 797. (Hagnard). Marine salt 200, Ammonium chlorid 90, Water 710. (Howen). Ordinary heavy mineral water bottles may be used.

---

**Coronary Thrombosis.** B. M. Randolph, of Washington, Wash. Med. Annals, Sept., reports a case of angina in a man of 40 appearing younger but having marked arteriosclerosis. Attacks had occurred at intervals for four years, following active exercise. No murmurs but heart enlarged and weak. History of moderate use of alcohol and of typhoid ten years before. Wassermann reaction positive. A clot was found in the right coronary, containing disorganized red cells, deposits of haemosiderin and beginning formation of fibrous tissue, indicating that it must have occurred some time before death.

